# 2-Amino-4-methylpyridinium 2-hy­droxy-3,5-dinitro­benzoate

**DOI:** 10.1107/S1600536810025912

**Published:** 2010-07-07

**Authors:** Ching Kheng Quah, Madhukar Hemamalini, Hoong-Kun Fun

**Affiliations:** a X-ray Crystallography Unit, School of Physics, Universiti Sains Malaysia, 11800 USM, Penang, Malaysia

## Abstract

In the anion of the title mol­ecular salt, C_6_H_9_N_2_^+^·C_7_H_3_N_2_O_7_^−^, the two nitro groups are twisted from the attached benzene ring with dihedral angles of 27.36 (10) and 4.86 (11)°. The anion is stabilized by an intra­molecular O—H⋯O hydrogen bond, which generates an *S*(6) ring motif. In the crystal, the cations and anions are linked by N—H⋯O and C—H⋯O inter­actions and are further consolidated by C—H⋯π inter­actions, to generate a three-dimensional network. A short O⋯N contact of 2.876 (2) Å also occurs.

## Related literature

For substituted pyridines, see: Pozharski *et al.* (1997 ▶); Katritzky *et al.* (1996 ▶). For details of hydrogen bonding, see: Scheiner (1997 ▶); Jeffrey & Saenger (1991 ▶); Jeffrey (1997 ▶). For 2-amino-substituted pyridines, see: Navarro Ranninger *et al.* (1985 ▶); Luque *et al.* (1997 ▶); Qin *et al.* (1999 ▶); Ren *et al.* (2002 ▶); Rivas *et al.* (2003 ▶); Jin *et al.* (2001 ▶); Albrecht *et al.* (2003 ▶). For Lewis bases with 3,5-dinitrosalicylic acid, see: Hindawey *et al.* (1980 ▶); Issa *et al.* (1981 ▶). For hydrogen-bond motifs, see: Bernstein *et al.* (1995 ▶). For related structures, see: Quah *et al.* (2008*a* ▶,*b* ▶, 2010 ▶). For reference bond lengths, see: Allen *et al.* (1987 ▶). For the stability of the temperature controller used for the data collection, see: Cosier & Glazer (1986 ▶).
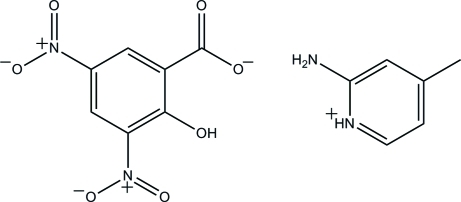


## Experimental

### 

#### Crystal data

C_6_H_9_N_2_^+^·C_7_H_3_N_2_O_7_^−^
*M* *_r_* = 336.27
Monoclinic, 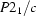

*a* = 6.0111 (15) Å
*b* = 9.652 (3) Å
*c* = 24.436 (6) Å
β = 100.546 (7)°
*V* = 1393.8 (7) Å^3^
*Z* = 4
Mo *K*α radiation
μ = 0.13 mm^−1^
*T* = 100 K
0.48 × 0.08 × 0.06 mm

#### Data collection

Bruker SMART APEXII DUO CCD diffractometer
Absorption correction: multi-scan (*SADABS*; Bruker, 2009 ▶) *T*_min_ = 0.939, *T*_max_ = 0.992
12178 measured reflections
3229 independent reflections
2283 reflections with *I* > 2σ(*I*)
*R*_int_ = 0.057

#### Refinement

*R*[*F* ^2^ > 2σ(*F* ^2^)] = 0.047
*wR*(*F* ^2^) = 0.153
*S* = 1.03
3229 reflections
222 parameters
H-atom parameters constrained
Δρ_max_ = 0.31 e Å^−3^
Δρ_min_ = −0.44 e Å^−3^

### 

Data collection: *APEX2* (Bruker, 2009 ▶); cell refinement: *SAINT* (Bruker, 2009 ▶); data reduction: *SAINT*; program(s) used to solve structure: *SHELXTL* (Sheldrick, 2008 ▶); program(s) used to refine structure: *SHELXTL*; molecular graphics: *SHELXTL*; software used to prepare material for publication: *SHELXTL* and *PLATON* (Spek, 2009 ▶).

## Supplementary Material

Crystal structure: contains datablocks global, I. DOI: 10.1107/S1600536810025912/hb5538sup1.cif

Structure factors: contains datablocks I. DOI: 10.1107/S1600536810025912/hb5538Isup2.hkl

Additional supplementary materials:  crystallographic information; 3D view; checkCIF report

## Figures and Tables

**Table 1:** Hydrogen-bond geometry (Å, °)

*D*—H⋯*A*	*D*—H	H⋯*A*	*D*⋯*A*	*D*—H⋯*A*
N1—H1*N*1⋯O6^i^	0.96	1.75	2.707 (2)	175
N2—H1*N*2⋯O7^i^	0.98	1.93	2.907 (2)	173
N2—H2*N*2⋯O1^ii^	0.95	2.25	2.974 (2)	133
N2—H2*N*2⋯O2^ii^	0.95	2.23	3.089 (2)	151
O1—H1*O*1⋯O7	0.99	1.60	2.426 (2)	138
C2—H2*A*⋯O2^ii^	0.93	2.54	3.300 (3)	139
C4—H4*A*⋯O6^iii^	0.93	2.53	3.447 (3)	169
C5—H5*A*⋯O5^iv^	0.93	2.37	3.193 (3)	147
C9—H9*A*⋯O3^v^	0.93	2.38	3.272 (3)	161
C6—H6*B*⋯*Cg*1^iii^	0.96	2.99	3.623 (2)	12

Symmetry codes: (i) 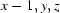; (ii) 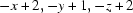; (iii) 
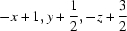
; (iv) 
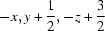
; (v) 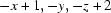.

**Fig. 1.**
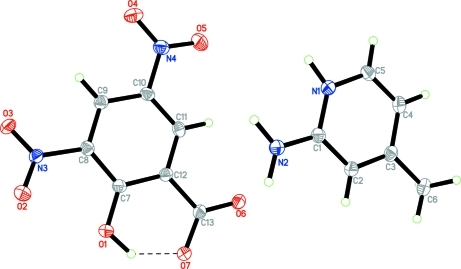
The molecular structure of (I) showing 50% probability displacement ellipsoids for non-H atoms. The intramolecular hydrogen bond is shown in dashed line.

**Fig. 2.**
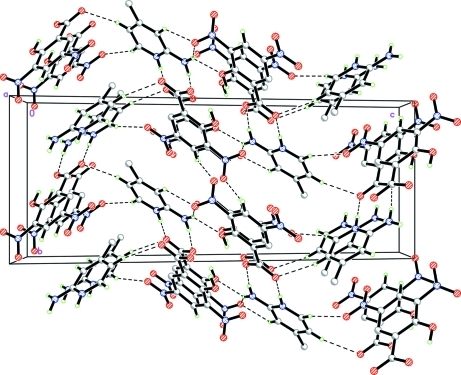
The crystal structure of (I) viewed along the a axis. H atoms not involved in intermolecular interactions (dashed lines) have been omitted for clarity.

## supplementary crystallographic information

### Comment

Pyridine and its derivatives play an important role in heterocyclic chemistry (Pozharski *et al.*, 1997; Katritzky *et al.*, 1996). They are often involved in hydrogen-bond interactions (Jeffrey & Saenger, 1991; Jeffrey, 1997; Scheiner, 1997). There are numerous examples of 2-amino-substituted pyridine compounds in which the 2-aminopyridines act as neutral ligands (Navarro Ranninger *et al.*, 1985; Qin *et al.*, 1999; Ren *et al.*, 2002; Rivas *et al.*, 2003) or as protonated cations (Luque *et al.*, 1997; Jin *et al.*, 2001; Albrecht *et al.*, 2003). The nitrosubstituted aromatic acid 3,5-dinitro salicylic acid (DNSA) has proven potential for formation of proton-transfer compounds, particularly because of its acid strength (p*K*_a_ = 2.18), its interactive *ortho*-related phenolic substituent group together with the nitro substituents which have potential for both π···π interactions as well as hydrogen-bonding interactions. A large number of both neutral and proton-transfer compounds of Lewis bases with DNSA, together with their IR spectra have been reported (Hindawey *et al.*, 1980; Issa *et al.*, 1981) in the literature. Since our aim is to study some interesting hydrogen bonding interactions, the crystal structure of the title compound is presented here.

The asymmetric unit of the title compound contains one 2-amino-4-methyl-pyridinium cation and one 3,5-dinitrosalicylate anion. A proton transfer from the carboxyl group of 3,5-dinitrosalicylic acid to atom N1 of 2-amino-4-methylpyridinium resulted in the formation of ions. The bond lengths (Allen *et al.*, 1987) and angles in the title compound (Fig. 1) are within normal ranges and comparable with the related structures (Quah *et al.*, 2010, 2008*a*, b). In the 3,5-dinitrosalicylate anion, the two nitro groups are twisted slightly from the attached ring. The dihedral angles between benzene ring (C8—C12) and the two nitro groups (O2/O3/N3/C8 and O4/O5/N4/C10) are 27.36 (10) and 4.86 (11)°, respectively. The 2-amino-4-methylpyridinium cation is essentially planar, with the maximum deviation of 0.012 (2) Å for atoms N2 and C4; and make a dihedral angle of 7.16 (8)° with the benzene ring of 3,5-dinitrosalicylate anion. The molecular structure is stabilized by an intramolecular O1–H1O1···O7 hydrogen bond which generates an *S*(6) ring motif (Bernstein *et al.*, 1995). There is a short O3···N3 contact (symmetry code: 2 - *x*, -*y*, 2 - *z*) with distance = 2.876 (2) Å which is shorter than the sum of van der Waals radii of the oxygen and nitrogen atoms.

In the crystal packing, the cations and anions are linked to form a three-dimensional network (Fig. 2) by intermolecular N1–H1N1···O6, N2–H1N2···O7, N2–H2N2···O1, N2–H2N2···O2, C2–H2A···O2, C4–H4A···O6, C5–H5A···O5 and C9–H9A···O3 interactions and are further consolidated by C–H···π (Table 1) interactions.

### Experimental

A hot methanol solution (20 ml) of 2-amino-4-methylpyridine (27 mg, Aldrich) and 3,5-dinitrosalicylic acid (57 mg, Merck) were mixed and warmed over a heating hotplate magnetic stirrer hotplate for a few minutes. The resulting solution was allowed to cool slowly to room temperature and yellow needles of (I) appeared after a few days.

### Refinement

O– and N-bound H atoms were located in a difference Fourier map and refined freely [O1—H1O1 = 0.9856 Å, N—H = 0.9478 - 0.9833 Å]. The remaining H atoms were positioned geometrically and refined using a riding model with C—H = 0.93–0.98 Å and *U*_iso_(H) = 1.2 or 1.5 *U*_eq_(C). A rotating-group model was applied for the methyl groups. The highest residual electron density peak is located at 0.79 Å from H6B and the deepest hole is located at 0.70 Å from N4.

### Crystal data

**Table:**

C_6_H_9_N_2_^+^·C_7_H_3_N_2_O_7_^−^	*F*(000) = 696
*M**_r_* = 336.27	*D*_x_ = 1.602 Mg m^−^^3^
Monoclinic, *P*2_1_/*c*	Mo *K*α radiation, λ = 0.71073 Å
Hall symbol: -P 2ybc	Cell parameters from 2122 reflections
*a* = 6.0111 (15) Å	θ = 2.7–26.4°
*b* = 9.652 (3) Å	µ = 0.13 mm^−^^1^
*c* = 24.436 (6) Å	*T* = 100 K
β = 100.546 (7)°	Needle, yellow
*V* = 1393.8 (7) Å^3^	0.48 × 0.08 × 0.06 mm
*Z* = 4	

### Data collection

**Table:**

Bruker SMART APEXII DUO CCD diffractometer	3229 independent reflections
Radiation source: fine-focus sealed tube	2283 reflections with *I* > 2σ(*I*)
graphite	*R*_int_ = 0.057
φ and ω scans	θ_max_ = 27.6°, θ_min_ = 1.7°
Absorption correction: multi-scan (*SADABS*; Bruker, 2009)	*h* = −7→7
*T*_min_ = 0.939, *T*_max_ = 0.992	*k* = −12→12
12178 measured reflections	*l* = −31→26

### Refinement

**Table:**

Refinement on *F*^2^	Primary atom site location: structure-invariant direct methods
Least-squares matrix: full	Secondary atom site location: difference Fourier map
*R*[*F*^2^ > 2σ(*F*^2^)] = 0.047	Hydrogen site location: inferred from neighbouring sites
*wR*(*F*^2^) = 0.153	H-atom parameters constrained
*S* = 1.02	*w* = 1/[σ^2^(*F*_o_^2^) + (0.0909*P*)^2^] where *P* = (*F*_o_^2^ + 2*F*_c_^2^)/3
3229 reflections	(Δ/σ)_max_ = 0.001
222 parameters	Δρ_max_ = 0.31 e Å^−^^3^
0 restraints	Δρ_min_ = −0.44 e Å^−^^3^

### Special details

**Table:**

Experimental. The crystal was placed in the cold stream of an Oxford Cyrosystems Cobra open-flow nitrogen cryostat (Cosier & Glazer, 1986) operating at 100.0 (1) K.
Geometry. All e.s.d.'s (except the e.s.d. in the dihedral angle between two l.s. planes) are estimated using the full covariance matrix. The cell e.s.d.'s are taken into account individually in the estimation of e.s.d.'s in distances, angles and torsion angles; correlations between e.s.d.'s in cell parameters are only used when they are defined by crystal symmetry. An approximate (isotropic) treatment of cell e.s.d.'s is used for estimating e.s.d.'s involving l.s. planes.
Refinement. Refinement of *F*^2^ against ALL reflections. The weighted *R*-factor *wR* and goodness of fit *S* are based on *F*^2^, conventional *R*-factors *R* are based on *F*, with *F* set to zero for negative *F*^2^. The threshold expression of *F*^2^ > σ(*F*^2^) is used only for calculating *R*-factors(gt) *etc*. and is not relevant to the choice of reflections for refinement. *R*-factors based on *F*^2^ are statistically about twice as large as those based on *F*, and *R*- factors based on ALL data will be even larger.

### Fractional atomic coordinates and isotropic or equivalent isotropic displacement parameters (Å^2^)

**Table:**

	*x*	*y*	*z*	*U*_iso_*/*U*_eq_	
N1	0.2405 (3)	0.80105 (17)	0.83755 (7)	0.0179 (4)	
H1N1	0.1325	0.7348	0.8455	0.034 (7)*	
N2	0.5190 (3)	0.75204 (18)	0.91264 (7)	0.0220 (4)	
H1N2	0.4146	0.6810	0.9219	0.052 (9)*	
H2N2	0.6655	0.7664	0.9340	0.063 (10)*	
C1	0.4517 (3)	0.8236 (2)	0.86605 (8)	0.0170 (4)	
C2	0.5856 (3)	0.9234 (2)	0.84489 (9)	0.0187 (4)	
H2A	0.7321	0.9408	0.8636	0.022*	
C3	0.5022 (3)	0.9946 (2)	0.79722 (9)	0.0187 (4)	
C4	0.2784 (3)	0.9680 (2)	0.76912 (9)	0.0199 (4)	
H4A	0.2179	1.0163	0.7369	0.024*	
C5	0.1541 (3)	0.8708 (2)	0.79013 (9)	0.0192 (4)	
H5A	0.0076	0.8518	0.7718	0.023*	
C6	0.6432 (4)	1.1006 (2)	0.77401 (9)	0.0231 (5)	
H6A	0.7790	1.1181	0.8006	0.035*	
H6B	0.5587	1.1850	0.7666	0.035*	
H6C	0.6820	1.0662	0.7401	0.035*	
O1	1.1381 (2)	0.34244 (14)	0.99102 (6)	0.0198 (3)	
H1O1	1.2304	0.4181	0.9798	0.119 (16)*	
O2	0.9779 (2)	0.16274 (14)	1.05842 (6)	0.0218 (4)	
O3	0.7607 (2)	0.00824 (15)	1.01170 (7)	0.0242 (4)	
O4	0.1674 (2)	0.17877 (16)	0.86962 (7)	0.0264 (4)	
O5	0.2470 (3)	0.33999 (17)	0.81463 (7)	0.0321 (4)	
O6	0.9513 (2)	0.60237 (15)	0.85954 (6)	0.0219 (3)	
O7	1.2134 (2)	0.53070 (14)	0.93122 (6)	0.0206 (3)	
N3	0.8463 (3)	0.12385 (17)	1.01644 (7)	0.0176 (4)	
N4	0.2953 (3)	0.26704 (18)	0.85650 (7)	0.0212 (4)	
C7	0.9355 (3)	0.32562 (19)	0.96014 (9)	0.0159 (4)	
C8	0.7863 (3)	0.2196 (2)	0.97011 (8)	0.0169 (4)	
C9	0.5784 (3)	0.1992 (2)	0.93658 (9)	0.0172 (4)	
H9A	0.4837	0.1279	0.9438	0.021*	
C10	0.5143 (3)	0.2876 (2)	0.89190 (8)	0.0176 (4)	
C11	0.6536 (3)	0.3935 (2)	0.88017 (9)	0.0179 (4)	
H11A	0.6066	0.4515	0.8499	0.022*	
C12	0.8626 (3)	0.4127 (2)	0.91368 (8)	0.0164 (4)	
C13	1.0164 (3)	0.5246 (2)	0.89969 (9)	0.0175 (4)	

### Atomic displacement parameters (Å^2^)

**Table:**

	*U*^11^	*U*^22^	*U*^33^	*U*^12^	*U*^13^	*U*^23^
N1	0.0180 (8)	0.0159 (8)	0.0193 (9)	−0.0010 (6)	0.0019 (7)	−0.0004 (7)
N2	0.0196 (8)	0.0221 (9)	0.0226 (10)	−0.0029 (7)	−0.0002 (7)	0.0060 (7)
C1	0.0167 (9)	0.0148 (9)	0.0197 (11)	0.0023 (7)	0.0034 (8)	−0.0016 (8)
C2	0.0175 (9)	0.0153 (9)	0.0229 (11)	0.0004 (7)	0.0029 (8)	0.0005 (8)
C3	0.0221 (10)	0.0133 (9)	0.0218 (11)	0.0011 (8)	0.0066 (8)	−0.0027 (8)
C4	0.0242 (10)	0.0159 (10)	0.0195 (11)	0.0034 (8)	0.0039 (8)	0.0006 (8)
C5	0.0181 (9)	0.0189 (10)	0.0195 (11)	0.0037 (8)	0.0008 (8)	−0.0012 (8)
C6	0.0278 (11)	0.0168 (10)	0.0256 (12)	0.0000 (8)	0.0069 (9)	0.0029 (9)
O1	0.0170 (7)	0.0188 (7)	0.0215 (8)	−0.0024 (6)	−0.0014 (6)	0.0008 (6)
O2	0.0205 (7)	0.0234 (8)	0.0200 (8)	−0.0013 (6)	−0.0001 (6)	−0.0001 (6)
O3	0.0203 (7)	0.0164 (7)	0.0353 (9)	−0.0040 (6)	0.0033 (6)	0.0049 (7)
O4	0.0200 (7)	0.0276 (8)	0.0309 (9)	−0.0066 (6)	0.0028 (6)	−0.0028 (7)
O5	0.0303 (9)	0.0338 (9)	0.0272 (9)	−0.0016 (7)	−0.0084 (7)	0.0069 (7)
O6	0.0245 (8)	0.0183 (7)	0.0216 (8)	−0.0039 (6)	0.0006 (6)	0.0028 (6)
O7	0.0182 (7)	0.0181 (7)	0.0244 (8)	−0.0021 (5)	0.0009 (6)	0.0025 (6)
N3	0.0152 (8)	0.0162 (8)	0.0218 (9)	−0.0002 (6)	0.0042 (7)	0.0022 (7)
N4	0.0187 (8)	0.0203 (9)	0.0238 (10)	0.0003 (7)	0.0016 (7)	−0.0026 (8)
C7	0.0144 (9)	0.0144 (9)	0.0186 (10)	0.0010 (7)	0.0021 (8)	−0.0025 (8)
C8	0.0186 (9)	0.0139 (9)	0.0177 (10)	0.0021 (7)	0.0020 (8)	−0.0006 (8)
C9	0.0169 (9)	0.0142 (9)	0.0207 (11)	−0.0004 (7)	0.0037 (8)	−0.0017 (8)
C10	0.0161 (9)	0.0162 (9)	0.0191 (11)	0.0008 (7)	−0.0007 (8)	−0.0028 (8)
C11	0.0198 (9)	0.0159 (9)	0.0181 (10)	0.0043 (8)	0.0035 (8)	0.0000 (8)
C12	0.0164 (9)	0.0132 (9)	0.0199 (11)	0.0005 (7)	0.0038 (8)	−0.0018 (8)
C13	0.0189 (9)	0.0144 (9)	0.0193 (11)	−0.0002 (7)	0.0040 (8)	−0.0001 (8)

### Geometric parameters (Å, °)

**Table:**

N1—C1	1.349 (2)	O1—H1O1	0.9856
N1—C5	1.359 (3)	O2—N3	1.234 (2)
N1—H1N1	0.9560	O3—N3	1.226 (2)
N2—C1	1.330 (3)	O4—N4	1.229 (2)
N2—H1N2	0.9833	O5—N4	1.232 (2)
N2—H2N2	0.9478	O6—C13	1.241 (2)
C1—C2	1.413 (3)	O7—C13	1.291 (2)
C2—C3	1.366 (3)	N3—C8	1.455 (3)
C2—H2A	0.9300	N4—C10	1.450 (2)
C3—C4	1.417 (3)	C7—C8	1.411 (3)
C3—C6	1.504 (3)	C7—C12	1.416 (3)
C4—C5	1.358 (3)	C8—C9	1.377 (3)
C4—H4A	0.9300	C9—C10	1.383 (3)
C5—H5A	0.9300	C9—H9A	0.9300
C6—H6A	0.9600	C10—C11	1.385 (3)
C6—H6B	0.9600	C11—C12	1.381 (3)
C6—H6C	0.9600	C11—H11A	0.9300
O1—C7	1.320 (2)	C12—C13	1.501 (3)
			
C1—N1—C5	122.49 (18)	O3—N3—O2	123.37 (17)
C1—N1—H1N1	127.8	O3—N3—C8	117.70 (16)
C5—N1—H1N1	109.6	O2—N3—C8	118.93 (16)
C1—N2—H1N2	116.8	O4—N4—O5	123.33 (18)
C1—N2—H2N2	120.1	O4—N4—C10	118.81 (17)
H1N2—N2—H2N2	122.9	O5—N4—C10	117.86 (17)
N2—C1—N1	117.93 (18)	O1—C7—C8	122.67 (18)
N2—C1—C2	124.26 (18)	O1—C7—C12	120.20 (18)
N1—C1—C2	117.80 (18)	C8—C7—C12	117.10 (17)
C3—C2—C1	120.67 (19)	C9—C8—C7	122.50 (19)
C3—C2—H2A	119.7	C9—C8—N3	116.15 (17)
C1—C2—H2A	119.7	C7—C8—N3	121.34 (17)
C2—C3—C4	119.33 (19)	C8—C9—C10	118.22 (19)
C2—C3—C6	121.29 (19)	C8—C9—H9A	120.9
C4—C3—C6	119.38 (18)	C10—C9—H9A	120.9
C5—C4—C3	118.77 (19)	C9—C10—C11	121.79 (18)
C5—C4—H4A	120.6	C9—C10—N4	118.56 (18)
C3—C4—H4A	120.6	C11—C10—N4	119.65 (18)
C4—C5—N1	120.93 (18)	C12—C11—C10	119.70 (19)
C4—C5—H5A	119.5	C12—C11—H11A	120.2
N1—C5—H5A	119.5	C10—C11—H11A	120.2
C3—C6—H6A	109.5	C11—C12—C7	120.68 (18)
C3—C6—H6B	109.5	C11—C12—C13	119.52 (18)
H6A—C6—H6B	109.5	C7—C12—C13	119.77 (17)
C3—C6—H6C	109.5	O6—C13—O7	124.53 (18)
H6A—C6—H6C	109.5	O6—C13—C12	119.82 (18)
H6B—C6—H6C	109.5	O7—C13—C12	115.65 (17)
C7—O1—H1O1	116.1		
			
C5—N1—C1—N2	178.61 (18)	N3—C8—C9—C10	−179.85 (17)
C5—N1—C1—C2	−0.4 (3)	C8—C9—C10—C11	0.6 (3)
N2—C1—C2—C3	−178.7 (2)	C8—C9—C10—N4	−179.81 (17)
N1—C1—C2—C3	0.2 (3)	O4—N4—C10—C9	5.3 (3)
C1—C2—C3—C4	0.3 (3)	O5—N4—C10—C9	−174.93 (18)
C1—C2—C3—C6	−179.65 (19)	O4—N4—C10—C11	−175.09 (18)
C2—C3—C4—C5	−0.8 (3)	O5—N4—C10—C11	4.7 (3)
C6—C3—C4—C5	179.17 (19)	C9—C10—C11—C12	0.0 (3)
C3—C4—C5—N1	0.7 (3)	N4—C10—C11—C12	−179.65 (18)
C1—N1—C5—C4	−0.1 (3)	C10—C11—C12—C7	−0.4 (3)
O1—C7—C8—C9	−177.55 (19)	C10—C11—C12—C13	177.93 (18)
C12—C7—C8—C9	0.3 (3)	O1—C7—C12—C11	178.16 (18)
O1—C7—C8—N3	1.6 (3)	C8—C7—C12—C11	0.3 (3)
C12—C7—C8—N3	179.39 (17)	O1—C7—C12—C13	−0.2 (3)
O3—N3—C8—C9	26.5 (3)	C8—C7—C12—C13	−178.04 (17)
O2—N3—C8—C9	−152.80 (18)	C11—C12—C13—O6	2.8 (3)
O3—N3—C8—C7	−152.64 (18)	C7—C12—C13—O6	−178.83 (19)
O2—N3—C8—C7	28.0 (3)	C11—C12—C13—O7	−176.29 (18)
C7—C8—C9—C10	−0.7 (3)	C7—C12—C13—O7	2.1 (3)

### Hydrogen-bond geometry (Å, °)

**Table:**

*D*—H···*A*	*D*—H	H···*A*	*D*···*A*	*D*—H···*A*
N1—H1N1···O6^i^	0.96	1.75	2.707 (2)	175
N2—H1N2···O7^i^	0.98	1.93	2.907 (2)	173
N2—H2N2···O1^ii^	0.95	2.25	2.974 (2)	133
N2—H2N2···O2^ii^	0.95	2.23	3.089 (2)	151
O1—H1O1···O7	0.99	1.60	2.426 (2)	138
C2—H2A···O2^ii^	0.93	2.54	3.300 (3)	139
C4—H4A···O6^iii^	0.93	2.53	3.447 (3)	169
C5—H5A···O5^iv^	0.93	2.37	3.193 (3)	147
C9—H9A···O3^v^	0.93	2.38	3.272 (3)	161
C6—H6B···Cg1^iii^	0.96	2.99	3.623 (2)	12

Symmetry codes: (i) *x*−1, *y*, *z*; (ii) −*x*+2, −*y*+1, −*z*+2; (iii) −*x*+1, *y*+1/2, −*z*+3/2; (iv) −*x*, *y*+1/2, −*z*+3/2; (v) −*x*+1, −*y*, −*z*+2.
